# Machine learning approaches for early detection of non-alcoholic steatohepatitis based on clinical and blood parameters

**DOI:** 10.1038/s41598-024-51741-0

**Published:** 2024-01-30

**Authors:** Amir Reza Naderi Yaghouti, Hamed Zamanian, Ahmad Shalbaf

**Affiliations:** 1 Department of Biomedical Engineering, Science and Research Branch, Islamic Azad University, Tehran, Iran; 2 Department of Biomedical Engineering and Medical Physics, School of Medicine, Shahid Beheshti University of Medical Sciences, Tehran, Iran

**Keywords:** Biomedical engineering, Image processing, Machine learning

## Abstract

This study aims to develop a machine learning approach leveraging clinical data and blood parameters to predict non-alcoholic steatohepatitis (NASH) based on the NAFLD Activity Score (NAS). Using a dataset of 181 patients, we performed preprocessing including normalization and categorical encoding. To identify predictive features, we applied sequential forward selection (SFS), chi-square, analysis of variance (ANOVA), and mutual information (MI). The selected features were used to train machine learning classifiers including SVM, random forest, AdaBoost, LightGBM, and XGBoost. Hyperparameter tuning was done for each classifier using randomized search. Model evaluation was performed using leave-one-out cross-validation over 100 repetitions. Among the classifiers, random forest, combined with SFS feature selection and 10 features, obtained the best performance: Accuracy: 81.32% ± 6.43%, Sensitivity: 86.04% ± 6.21%, Specificity: 70.49% ± 8.12% Precision: 81.59% ± 6.23%, and F1-score: 83.75% ± 6.23% percent. Our findings highlight the promise of machine learning in enhancing early diagnosis of NASH and provide a compelling alternative to conventional diagnostic techniques. Consequently, this study highlights the promise of machine learning techniques in enhancing early and non-invasive diagnosis of NASH based on readily available clinical and blood data. Our findings provide the basis for developing scalable approaches that can improve screening and monitoring of NASH progression.

## Introduction

The global prevalence of non-alcoholic fatty liver disease (NAFLD) is considerable: according to a recent meta-analysis, it affects 25.24% of the general population worldwide and is associated with an increased risk of liver-related and cardiovascular mortality. This makes NAFLD a widespread and serious health concern^1^. Moreover, NAFLD can progress and serve as a precursor to more severe liver conditions, including cirrhosis and cancer^2^. In advanced stages, NAFLD can progress to non-alcoholic steatohepatitis (NASH), characterized by hepatic steatosis, inflammation, hepatocyte injury (ballooning), and frequently, fibrosis^3^. It is estimated that 20 to 30% of people with NAFL will develop NASH, and if not treated on time, this condition can lead to cirrhosis of the liver and, in the most severe cases, hepatocellular carcinoma and an increase in cardiovascular diseases^4^. Consequently, early detection of NASH is critical to prevent the risks affiliated with its advancement. In parallel, regular monitoring of a patient’s condition can considerably lower the financial burdens associated with the treatment.

Although liver biopsy is considered the gold standard for the diagnosis of NASH, it is an invasive procedure that can lead to serious complications such as internal bleeding or even mortality rate of 0.01% in cases. Moreover, the incidence of major bleeding after liver biopsy varies widely, from 0 to 5.3%, depending on the source and the method of biopsy. Additionally, liver biopsy can cause pain in up to 84% of patients, which may require analgesic treatment. As a result, it reduces its routine applications^5,6^. Therefore, noninvasive diagnostic methods, including ultrasound, computed tomography (CT) scans, medical magnetic resonance imaging (MRI), and patient demographics have been developed^7,8^. However, these alternatives also have limitations as their accuracy depends heavily on the skill and expertise of the analysts examining the images, potentially affecting the accuracy of diagnosis. A more practical and popular non-invasive approach to diagnosing NASH involves utilizing clinical data and patient laboratory test results, including blood tests. This type of data is typically easy to obtain and collect without major discomfort to patients. Hence, these data sources are crucial tools in NASH diagnosis and analysis^9^.

Machine learning models, drawing on clinical and laboratory data, are now being explored as tools for the diagnosis and classification of diseases. The correlation of related features in different stages of the disease can lead to inaccurate assessments for an individual, potentially causing undue distress and unnecessary expenses. Nonetheless, by leveraging prior learning from similar situations, intelligent algorithms can be used rapidly and confidently, to provide reliable estimations as an assistant for the specialist. The physicians will be able to make complementary decisions based on them. Various studies have employed machine learning algorithms using clinical information to diagnose fatty liver diseases using. Ma et al. leveraged numerous features such as aspartate transaminase (AST); alanine transaminase (ALT); glutamic pyruvic transaminase (GPT); high-density lipoprotein (HDL); low-density lipoprotein (LDL); cholesterol; and glyceride to classify NAFLD^10^. They implemented algorithms in tenfold cross validation, like logistic regression (LR), k-nearest neighbors (kNN), support vector machine (SVM), adaptive boosting (AdaBoost), random forest (RF), and decision tree (DT). In this context, the LR algorithm emerged as the most effective for the intended classification in accuracy and SVM achieved the best performance in specificity and precision. Techniques for feature selection were employed to eliminate superfluous features. In a different study, Wu et al. deployed smart learning algorithms for fatty liver estimation based on clinical data^11^. They analyzed a dataset, including demographic details and patients' blood parameters of total 577 patients. After data preprocessing and implementing feature forward selection, they were classified with RF, naive Bayes, neural network, and LR algorithms. Parameters like sex, age, systolic and diastolic pressure, glucose, triglyceride, HDL, AST, and ALT were utilized as features for classification. In their survey, RF showed the most appropriate performance in accuracy and AUC. Canbay scrutinized the efficacy of using clinical data to gauge the severity of NAFLD and NASH^12^. Among 164 collected training data of obese individuals, the features were selected by ensemble feature selection (EFS) to predict the histological NAFLD Activity Score (NAS score) According to LR model, the performance of these data was assessed by AUC of 0.73. Newsome developed an LR model for the estimation of low NASH and high fibrosis and used a set of blood test features and FibroScan^13^. The findings were confirmed by a database containing pathological assessments. Aravind used clinical data from a collection of candidates to develop a machine-learning model to estimate the SAF score based on steatosis, activity score, and fibrosis assessments^14^. The employed model was a multilayer perceptron model (MLP), trained on 16 different datasets. Comparatively, the results of using RF and LR models for fibrosis severity estimation were presented, with the MLP yielding superior performance^15^. Okanoue used blood test results and demographic features in discerning NAFLD and NASH levels^16^. They used 11 features derived from 324 participants. The gold standard labels of this data were achieved by two expert histopathologists. A neural network model was employed for this purpose which presents an appropriate performance in evaluation with and without considering fibrosis. Careterro proposed a mechanism to classify data collected from 1525 patients to diagnose NASH^17^. They reviewed electronic health records, utilizing 37 features for classification via the RF method. Despite the class imbalance, they used weighting and sampling techniques to mitigate this limitation. This approach can provide the accuracy performance in range of 79–87% to diagnose NASH. Ghandian presented a comparison of the performance of different machine learning algorithms to detect the progression of NAFL to NASH^18^. He conducted a study on the electronic health record (HER) information of 700 volunteers. The best features were selected, and an extreme gradient boosting classifier (XGBoost) was used for this study. The XGBoost classifier outperformed the capability of prediction from NAFL to NASH in AUC of 0.79, and 0.87 for fibrosis. Zamanian et al.^19^ used machine learning methods to diagnose NASH disease based on clinical data and blood factors. They compared different classifier algorithms along with various features selection approaches on 176 candidates. According to this, using the MRMR algorithm and RF method achieved higher accuracy, AUC, precision, and recall. Their evaluation was confirmed by McNemar test to compare the performance of different algorithms in achieving the correct labels.

In this study, our objective is to utilize supervised learning techniques for precise NASH prediction based on the NAS Score. Our investigation presents several significant advancements over previous NASH diagnostic approaches. While previous studies have delved into machine learning applications for disease classification, many have been constrained by the utilization of a limited number of classifiers in their analyses. Our research marks a pioneering improvement in utilizing an expansive range of classifiers. This inclusive approach spans across both linear models, such as LR and SVM, and non-linear algorithms, including AdaBoost, LightGBM, RF, and XGBoost. This diverse selection of classifiers significantly enriches our analysis, allowing for a more comprehensive exploration and interpretation of the complex relationships within the clinical and blood factor data. Furthermore, in contrast to conventional approaches that often adopt default settings or limited optimization, our study embarks on an extensive hyperparameter tuning journey using randomized search for each algorithm. In this study, we employ robust cross-validation with repeated leave-one-out over 100 repetitions, a rigorous evaluation strategy that minimizes the impact of potential overfitting. Moreover, our feature selection procedure incorporates four techniques, namely, Sequential Forward Selection (SFS), Analysis of variance (ANOVA), mutual information (MI), and chi-squared test. Additionally, we deviate from the traditional binary classification approach and venture into multi-class classification of NAS scores, aiming to accurately discern the severity of NASH rather than simply identifying its presence or absence. Finally, our research introduces a comprehensive comparison of various feature selection and classification methodologies, offering a more extensive and nuanced exploration than studies limited to a few techniques. This multifaceted analysis aims to identify the most optimal machine learning pipeline for NASH diagnosis. Consequently, our main goal is to identify the optimal combination of these feature selection methods and classifiers that provides the highest accuracy in estimating the NAS, which is a composite score based on steatosis, inflammation, and ballooning, a critical metric in diagnosing NASH. Secondly, we aim to develop an algorithm capable of predicting NAS and hence diagnosing NASH, relying solely on available clinical data and blood factors. By eliminating the need for invasive diagnostic methods such as liver biopsy, our proposed solution seeks to facilitate early diagnosis, thereby minimizing associated risk factors and potentially reducing the financial burden of treatment.

## Materials and methods

### Dataset

The patient pool for this investigation consisted of 176 individuals diagnosed with NAFLD, who were under the care of Ehime University Hospital^5^. Patients in the age group of 20 to 79 years were divided into 81 males and 100 females. A strict set of inclusion criteria was followed on this data: an ultrasonography or CT scan confirmation of liver issues, enzyme examination, an absence of complications due to other health conditions, a clean record of daily alcohol consumption, and no history or evidence of liver functional disorders or hepatocellular carcinoma (HCC). The study collected data from the remaining patients with four indicators (steatosis, fibrosis, inflammation, ballooning) and NAS Score results extracted from the pathological specimens. The data collection process was overseen and approved by the ethics board of Ehime University Hospital (approval ID number: 1012004, 1709008; University Hospital Medical Information Network ID: UMIN000010659, UMIN 000030222). All methods were carried out in accordance with relevant guidelines and regulations. Informed consent was obtained for all subjects. All the participants were treated with laparoscopic surgery, followed by the preparation and examination of their liver biopsy samples by two qualified pathologists who were not privy to any other patient-specific information. The NAS Score, calculated as the non-weighted sum of relevant indicators steatosis rated from 0 to 3, lobular inflammation rated from 0 to 3, and ballooning rated from 0 to 2, was determined based on the guidelines proposed by^20^.

In this study, we utilized a comprehensive set of data, stratified into two principal clusters for analysis. The first category, clinical data, including patient characteristics such as age, gender, and Body Mass Index (BMI). Additionally, a record of patients' usage of specific medications is included within this category. This comprises the application of statins and an array of other pharmaceutical substances intended for the management of cholesterol and triglycerides. These include, but not limited to, Colestilan, in the treatment of hyperphosphatasemia and hypercholesterolemia; Ezetimibe, a renowned blood cholesterol management drug; Fibrate, aim to reduce cholesterol and triglyceride levels; Eicosapentaenoic acid (EPA), an Omega-3 fatty acid; and lipid-lowering agents, signifying the use of any pharmaceuticals for cholesterol reduction, blood fat reduction, or anti-fatty blood treatment.

The second category, blood test parameters, includes the γ-GTP, Glutamate-Pyruvate Transaminase (GPT), Creatinine (Cre), HbA1c, Total cholesterol level (TC), Total level of triglyceride (TG), High-density lipoprotein (HDL), Low-density lipoprotein (LDL), and Lipoprotein level (Lp(a)) present in the blood serum. Each of these parameters is integral to understanding the multifaceted nature of the disease being examined. Table 1 represents a summary of the characteristics of clinical data and blood parameters used in this paper. In this table, the range of distribution of any variable has been categorized based on the NAS score definition on mean value ± standard deviation.

**Table 1:** Summary of characteristics of clinical data and blood parameter.

Feature	Describe	NAS score	Value/mean ± std
Sex (men/women)	–	–	–
		–	–
		–	–
Age (year)	–	0	54.65 ± 14.96 y
		1	59.59 ± 13.63 y
		2	52.36 ± 13.34 y
BMI (kg/m^2^)	Body Mass Index	0	25.71 ± 3.82 kg/m^2^
		1	27.63 ± 5.5 kg/m^2^
		2	29.11 ± 4.85 kg/m^2^
Lipid-lowering agents (Yes/No)	Medications used to reduce levels of unwanted cholesterol in the blood	–	–
		–	–
		–	–
Statin/ezetimibe/colestilan/fibrate/EPA	–	–	–
		–	–
		–	–
GPT	Glutamate-pyruvate transaminase, an enzyme found in the liver	0	42.98 ± 27.88 IU/L
		1	75.29 ± 51.56 IU/L
		2	106.37 ± 55.43 IU/L
γ-GTP	Gamma-glutamyl transferase, an enzyme to indicate liver function	0	83.0 ± 106.64 IU/L
		1	83.42 ± 73.08 IU/L
		2	97.08 ± 84.93 IU/L
Cre SI (µmol/L)	Creatinine Serum Index, indicating kidney function	0	67.42 ± 14.71 µmol/L
		1	64.51 ± 16.19 µmol/L
		2	62.51 ± 16.17 µmol/L
${HbA1}_{c}$	Glycated hemoglobin, average blood sugar levels over the past 2–3 months	0	6.32 ± 1.3%
		1	6.47 ± 1.09%
		2	7.04 ± 1.88%
TC SI (mmol/L)	Total Cholesterol Serum Index	0	4.96 ± 1.22 mmol/L
		1	4.74 ± 0.88 mmol/L
		2	5.31 ± 0.99 mmol/L
TG SI (mmol/L)	Triglycerides Serum Index, a type of fat found in the blood	0	1.34 ± 0.66 mmol/L
		1	1.47 ± 0.6 mmol/L
		2	2.01 ± 1.16 mmol/L
LDL SI (mmol/L)	Low-density lipoprotein Serum Index, "bad" cholesterol	0	3.01 ± 1.02 mmol/L
		1	2.95 ± 0.83 mmol/L
		2	3.35 ± 0.88 mmol/L
HDL SI (mmol/L)	High-density lipoprotein Serum Index, "good" cholesterol	0	1.32 ± 0.48 mmol/L
		1	1.14 ± 0.26 mmol/L
		2	1.16 ± 0.24 mmol/L
Lp(a) mg/dL	Lipoprotein(a), a type of cholesterol linked to a higher risk of heart disease	0	24.83 ± 29.39 mg/dL
		1	11.19 ± 9.04 mg/dL
		2	11.98 ± 13.89 mg/dL

The NAS scores in our dataset were categorized into 3 classes: 0 (scores ≤ 3), 1 (3 < scores ≤ 5), and 2 (scores > 5). Gender distribution showed 26 men with NAS score 0, 39 with score 1, and 35 with score 2, along with 22 women with score 0, 30 with score 1, and 24 with score 2. Moreover, lipid-lowering agents were used by 13 individuals with NAS score 0, 21 individuals with NAS score 1, and 16 individuals with NAS score 2. Additionally, the medication usage profile consisted of 45 individuals using Statin, 6 using Ezetimibe, 0 using Colestilan, 2 using fibrate, and 1 using EPA.

### Preprocessing

Data preprocessing plays a pivotal role in enhancing the quality and applicability of data in any analytical project. In our study, we executed a series of preprocessing procedures on the NASH dataset to make it ready for further analysis. Initially, we normalized continuous variables, such as age, BMI, LDL levels, Lp(a) levels, total cholesterol, GPT, and high-density lipoprotein cholesterol, to a standardized range between 0 and 1. Additionally, we converted binary variables, such as statin, ezetimibe, fibrate, EPA, lipid lowering agents, and sex to 0 and 1, ensuring consistency and compatibility in our dataset. The NAS score, which we used as the primary label for our dataset, was categorized as follows: scores up to 3 were labeled as 0, scores greater than 3 but up to 5 were labeled as 1, and scores exceeding 5 were labeled as 2. This categorization provides a systematic approach to predict the severity of the NAS condition, aiding in clearer interpretation and decision-making in the subsequent analysis stages^21,22^. The distribution of NAS scores in the dataset is as follows: NAS 0 (48 instances), NAS 1 (69 instances), and NAS 2 (59 instances).

### Feature selection

Feature selection is an essential step in building machine learning models, as it aims to improve the performance and efficiency of the models by reducing the dimensionality of the feature space. This step involves selecting the most relevant and informative features from a large set of features, and discarding the redundant ones^23^. In this study we adopted four distinct methodological paradigms for this purpose: SFS, ANOVA, MI, and the chi-squared (χ^2^) test. The selection of these methodologies was based on their inherent capabilities to identify salient features from our dataset, which in turn would augment the precision and elucidative capacity of the model we intended to develop. SFS follows an iterative methodology, commencing with an empty set of features and gradually introducing those that exhibit the most substantial enhancements in the model's predictive accuracy at each step. This methodical process ensures that the feature set derived is meticulously curated, devoid of extraneous or non-contributory elements. This precision is paramount as it serves to optimize the model's overall performance. SFS operates as a discerning filter, allowing only the most informative features to be integrated into the analysis. This meticulous approach is conducive to enhancing the precision and efficiency of the model, aligning it more closely with the underlying data patterns, and ultimately strengthening the validity of our analytical results^24^. After the SFS process, we applied the ANOVA^25^ to ascertain the statistical significance of categorical variables within our dataset. ANOVA facilitates the comparison of variances both intra-group; and inter-group for a given categorical variable, thereby elucidating its effect on the dependent variable. Variables that manifested a p-value below a predetermined threshold, usually 0.05, in the ANOVA assessment were deemed statistically significant and thus retained for further analysis. ANOVA can be expressed as follows:

1 $$F=\frac{Between-group \; variability}{Within-group \; variability}$$

where F is the test statistic that follows an F-distribution under the null hypothesis that all group means are equal.

In tandem with ANOVA, we enriched our feature selection methodology by incorporating the chi-squared (${\upchi }^{2}$) test, also recognized as Pearson's chi-squared test^26^, to evaluate the independence of categorical variables. This non-parametric statistical test plays a vital role in assessing the independence of categorical variables within our dataset. Its primary objective is to determine whether the observed frequencies within a contingency table are consistent with the expected frequencies. By doing so, it provides valuable insights into the significance of categorical features in relation to the target variable. The chi-squared test is a widely employed statistical technique that can be calculated as follows:

2 $${\upchi }^{2}=\sum_{i=1}^{n}\frac{{({O}_{i}-{E}_{i})}^{2}}{{E}_{i}}$$

where ${O}_{i}$ are the observed frequencies, ${E}_{i}$ are the expected frequencies under the null hypothesis of independence, and n is the number of cells in the contingency table.

In our final analytical step, we employed MI to measure how much the individual features and the target variable depend on each other or how much the uncertainty decreases. Distinct from correlation coefficients, which are limited to linear relationships, MI provides insights into all potential relationships, irrespective of their linearity. Features that exhibited elevated mutual information values were considered indispensable for our predictive endeavors, given their substantial information overlap with the target variable^27,28^. MI can be defined as follows:

3 $$I\left(X;Y\right)=\sum_{x\in X}\sum_{y\epsilon Y}p\left(x,y\right)log\frac{p(x,y)}{p\left(x\right)p(y)}$$

where X and Y are two random variables, $p\left(x,y\right)$ is their joint probability distribution, and $p\left(x\right)$ and p(y) are their marginal probability distributions.

By employing different types of feature selection methods, we were able to obtain a comprehensive perspective of the distinguishing features for Nash rate classification. This multi-method approach increased our measurement accuracy significantly, underscoring the value of various feature selection techniques in our analysis.

### Classification

In this study, our main goal was to leverage the power of supervised learning algorithms with the primary objective of accurately predicting NASH using the NAS score. For this task, we applied an array of well-established models, such as SVM, RF, AdaBoost, LightGBM, and XGBoost. We strategically selected these models to leverage the strengths of linear, non-linear, and ensemble techniques, ensuring a comprehensive and robust approach to model development. To optimize the performance of each classifier, we incorporated an automatic hyperparameter tuning process using a random search approach within a predefined range of hyperparameters for each classifier. This systematic exploration of hyperparameter space allowed us to seek the optimal configuration that maximized predictive accuracy. This hyperparameter tuning step was essential to fine-tune the models and enhance their predictive capabilities for our specific task^29^. In Table 2, we detail the specific hyperparameter tuning ranges and configurations for each classifier, ensuring a comprehensive and transparent approach to fine-tuning our models for maximum predictive accuracy. This table presents the essential hyperparameter of any given algorithm, along with the specified range of values to identify the optimal estimator. The determination of these ranges for hyperparameters was made based on the capabilities of the assumed algorithms, the maturity of the target data, and the computational power of our processors. According this, the hyperparameters with higher priority on the performance of the algorithms were assumed.

**Table 2:** Hyperparameter ranges for various machine learning classifiers.

Classifiers	Parameters	Range
Logistic regression	Regularization strength	$[0.001, 0.01, 0.1, 1, 10, 100]$
SVM	Kernel	Linear, RBF, polynomial
	Regularization strength	$[0.001, 0.01, 0.1, 1, 10, 100]$
Random forest	Tree depth	$[1, 5, 10, 15, 20]$
	Learning rate	$[0.01, 0.05, 0.1, 0.2]$
	Number of estimators	$[50, 100, 200, 300]$
AdaBoost	Tree depth	$[1, 2, 3, 4, 5]$
	Learning rate	$[0.01, 0.05, 0.1, 0.2]$
	Number of estimators	$[50, 100, 200, 300]$
LightGBM	Tree depth	$[1, 5, 10, 15, 20]$
	Learning rate	$[0.01, 0.05, 0.1, 0.2]$
	Number of estimators	$[50, 100, 200, 300]$
XGBoost	Tree depth	$[1, 5, 10, 15, 20]$
	Learning rate	$[0.01, 0.05, 0.1, 0.2]$
	Number of estimators	$[50, 100, 200, 300]$

We initiated our modeling with the SVM model, renowned for its flexibility in defining hyperplanes in a multidimensional space to segregate classes^30^. We also employed ensemble methods, such as Random Forest, AdaBoost, LightGBM, and XGBoost. A Random Forest is an ensemble of decision trees that reduces overfitting by introducing randomness and diversity in the tree construction. AdaBoost, on the other hand, assigns higher weights to the instances that are harder to classify, iteratively improving the overall accuracy. LightGBM adopts a histogram-based method to find the optimal split point for each node of the tree, which reduces the number of candidates split points by grouping the feature values into discrete bins. This method can significantly lower the computational cost and memory usage, as well as handle categorical features and missing values more effectively than GBM^31^. Lastly, XGBoost^32^, is a high-performance method that offers speed, scalability, and regularization parameters to prevent overfitting and improve generalization, forming an integral part of our classification techniques, and further fortifying our model development process.

### Statistical evaluation

In this study, we employed the repeated leave-one-out cross-validation method with 100 repetitions to evaluate the performance of different classification algorithms. In each repetition, we iteratively left out one data point from the training set and used the remaining data for training. This process was repeated 100 times with different leave-one-out splits. The results were calculated as the mean and standard deviation of the performance metrics, including accuracy, sensitivity (or recall), precision, and F1-score, across ten independent sets of the 100 repetitions. This approach allowed us to ensure robust and reliable assessment of the classification algorithms' performance, considering both the variability introduced by the repeated leave-one-out cross-validation and the consistency observed across multiple sets of these repetitions.

Accuracy is the ratio of correctly predicted instances to the total instances in the dataset.

4 $$Accuracy= \frac{Number \; of \;Correctly \; Predicted \; Instances}{Total \; Number \; of \; Instances}$$

Sensitivity or Recall is the ratio of correctly predicted positive labels to the total actual positive labels.

5 $$\mathrm{Sensitivity }= \frac{True \; Positives}{True\; Positives+ False \; Negatives}$$

Precision is the ratio of correctly predicted positive labels to the total number of labels that were predicted as positive.

6 $$\mathrm{Precision }= \frac{True \; Positives}{True\; Positives+ False \; Positives}$$

F1-score is the harmonic mean of accuracy and sensitivity.

7 $${\text{F-}}1 \; \mathrm{score }= \frac{2\times {\text{Sensitivity}}\times {\text{Precision}}}{Sensitivity+Precision}$$

### Overall investigation of the proposed algorithm

**Figure 1:**
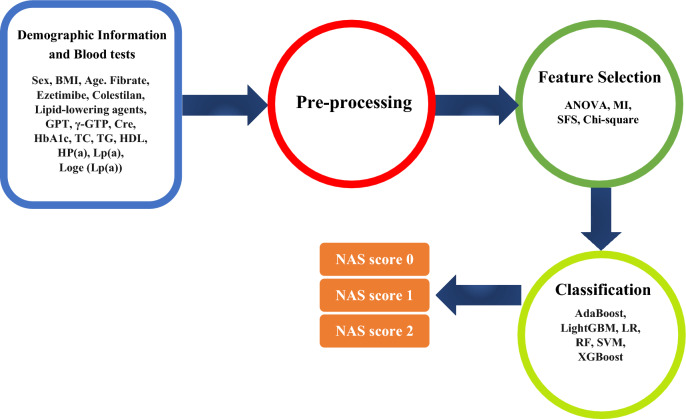
An overall schematic of the algorithm designed for NAS score prediction.

Figure 1 illustrates our methodology in detail. From the dataset, nineteen attributes were isolated to assess liver health. Initially, demographic details and blood tests were adjusted to facilitate value comparisons. These values underwent preprocessing steps including categorization, normalization, and binarization. We then applied various feature selection techniques such as SFS, Chi-square, ANOVA, and MI to identify the optimal set of features. The selected features were then classified using sophisticated algorithms, including SVM, RF, AdaBoost, LightGBM, and XGBoost. We reported the performance metrics of each algorithm, including accuracy, precision, recall, specificity, and f1-score. We measured the peak performance of each algorithm based on repeated leave-one-out cross-validation, repeated ten times for reliable results. We implemented our methodology in Python using Spyder, powered by an AMD Ryzen 7 6800H, with 16 GB RAM and an Nvidia RTX 3060 GPU.

## Results

Table 3 summarizes the diagnostic results of NASH obtained by different classification algorithms, showcasing the performance metrics with different feature selection methods and optimal numbers of features for each classifier. For each classifier, the table identifies the best feature selection method based on its accuracy and shows the corresponding number of selected features. Additionally, the table provides detailed performance metrics, including accuracy, precision, recall, specificity, and f1-score for a comprehensive evaluation. The results indicate that each classifier when paired with its respective best feature selection approach, achieves similar performance. The highest accuracy, based on the given data, was achieved by applying the SFS feature selection method and the RF classifier together. The SFS method selected 10 features based on their significance across different classes. The RF classifier used these features to predict NASH disease with an accuracy of $81.32 \pm 6.43$ based on repeated leave-one-out cross-validation.

**Table 3:** Performance metrics of various classification models (AdaBoost, LightGBM, LR, RF, SVM, and XGBoost) with different feature selection methods (chi-squared, SFS, ANOVA, and MI) and optimal numbers of features.

Classification	Feature selection	Number of features	Accuracy	Recall	Specificity	Precision	F1-score
AdaBoost	Chi-squared	15	74.83$\pm$7.32	$79.21 \pm 7.48$	59.45 ± 8.90	75.33 ± 7.71	77.24 ± 7.52
	SFS	8	79.36 ± 7.74	82.26$\pm \; 6.27$	65.17 ± 7.06	79.98$\pm \; 6.31$	81.10 ± $6.29$
	ANOVA	10	79.93 ± 7.67	83.41 ± 6.14	64.62 ± 7.82	80.17 ± 6.22	81.22 ± 6.18
	MI	10	$78.07\pm 7.53$	82.92 ± 6.60	63.97 ± 8.05	78.44 ± 7.06	80.62 ± $6.76$
	All Features		75.29$\pm$7.78	78.86 ± 7.34	60.14 ± 8.46	76.18 ± 7.56	77.56 ± 7.47
LightGBM	Chi-squared	15	75.38 ± 7.43	79.43 ± 7.73	62.07 ± 9.13	76.11 ± 7.08	77.75 ± 7.42
	SFS	10	80.53 ± 6.15	85.29 ± 5.81	68.92 ± 7.94	81.31 ± 6.07	83.25 ± 5.91
	ANOVA	15	78.41 ± 6. 88	82.50 ± 6.52	65.69 ± 8.22	79.09 ± 6.89	80.76 ± 5.64
	MI	12	79.61 ± 6.73	84.85 ± 6.16	66.12 ± 8.59	80.45 ± 6.52	82.59 ± 6.64
	All features		74.22 ± 7.50	79.04 ± 7.65	61.54 ± 9.60	74.89 ± 7.26	76.92 ± 7.48
LR	Chi-squared	15	72.36 ± 7.41	76.23 ± 6.60	59.94 ± 10.31	72.79 ± 6.98	73.47 ± 6.51
	SFS	10	76.64 ± 7.37	81.74 ± 6.98	64.16 ± 8.86	77.08 ± 7.11	79.35 ± 7.05
	ANOVA	10	76.08 ± 7.46	80.95 ± 7.39	63.89 ± 8.70	76.52 ± 6.72	78.69 ± 7.04
	MI	12	75.48 ± 7.10	80.91 ± 7.07	62.58 ± 9.12	76.16 ± 6.02	78.46 ± 7.51
	All features		71.91 ± 7.61	75.19 ± 7.12	57.30. 10.74	72.12 ± 7.52	77.26 ± 7.38
RF	Chi-squared	17	$76.65 \pm 7.44$	$81.33 \pm 7.99$	$64.08 \pm 9.22$	$76.92 \pm 7.43$	$79.06 \pm 7.53$
	**SFS**	**10**	$81.32\boldsymbol{ }\pm \boldsymbol{ }6.43$	$86.04\boldsymbol{ }\pm \boldsymbol{ }6.21$	$70.49\boldsymbol{ }\pm \boldsymbol{ }8.12$	$81.59\boldsymbol{ }\pm \boldsymbol{ }6.23$	$83.75\boldsymbol{ }\pm \boldsymbol{ }6.23$
	ANOVA	10	$80.03 \pm 6.22$	$85.38 \pm 6.48$	$69.50 \pm 8.31$	$80.42 \pm 6.62$	$82.82 \pm 6.28$
	MI	12	79.36 ± 6.43	83.47 ± 6.02	60.93 ± 8.69	79.74 ± 6.59	81.56 ± 6.34
	All features		$75.68 \pm 7.03$	$80.01 \pm 6.63$	$66.93 \pm 9.49$	$76.28 \pm 7.10$	$78.10 \pm 6.82$
SVM	Chi-squared	15	73.84$\pm$8.12	77.53$\pm \; 7.89$	59.73$\pm 11.04$	73.09$\pm \; 7.57$	75.24 ± 7.70
	SFS	12	$78.76\pm 7.72$	$83.08 \pm 7.58$	$63.66 \pm 8.58$	$78.84 \pm 6.63$	$81.21\pm 6.74$
	ANOVA	12	76.81$\pm$7.92	80.16$\pm$7.46	61.66$\pm$8.92	77.19$\pm$8.25	78.64$\pm$7.67
	MI	15	77.12$\pm \; 7.88$	81.81$\pm \; 7.67$	63.12$\pm \; 9.01$	77.20$\pm \; 6.91$	78.96$\pm$7.41
	All features		74.42$\pm$8.01	79.61$\pm$7.72	60.98$\pm \; 9.46$	75.41$\pm \; 7.13$	77.38$\pm$7.31
XGBoost	Chi-squared	17	76.93$\pm$7.53	81.72$\pm$7.08	61.86$\pm$10.62	78.08$\pm$7.49	79.85$\pm$7.29
	SFS	8	80.07$\pm \; 7.27$	85.09$\pm$6.74	70.42$\pm \; 7.94$	80.53$\pm \; 7.32$	82.74$\pm \; 7.14$
	ANOVA	10	$81.31 \pm 6.62$	$85.82 \pm 5.81$	$69.05 \pm 7.59$	$81.54 \pm 6.52$	$83.62\pm 6.64$
	MI	15	$77.90 \pm 7.18$	$82.40 \pm 6.96$	$67.83 \pm 9.55$	$78.29 \pm 7.09$	$80.29 \pm 7.03$
	All features		76.74 ± 7.78	81.64 ± 7.12	61.79 ± 9.72	77.98 ± 7.56	79.61 ± 7.34

Significant values are in bold.

Feature selection methods SFS and ANOVA exhibited distinct performance across different classifiers. Across a diverse set of classification algorithms, a consistent trend emerged revealing SFS outperforming ANOVA, except in the case of XGBoost classifier. The comparative analysis across classifiers demonstrates a recurrent advantage of SFS over ANOVA in optimizing feature selection for improved predictive performance. The Chi-squared feature selection method consistently demonstrated inferior performance compared to other feature selection techniques across a diverse range of classifiers, thereby highlighting its limited efficacy in achieving optimal predictive accuracy within this context. Additionally, a competitive landscape emerged between two classifiers, RF and XGBoost, showcasing their superior performance in comparison to others. Notably, LR consistently underperformed other classifiers across all metrics among various the feature selection methodologies. The highest achieved accuracy for LR was 76.64 ± 7.37, attained with 10 features selected via the SFS technique.

**Figure 2:**
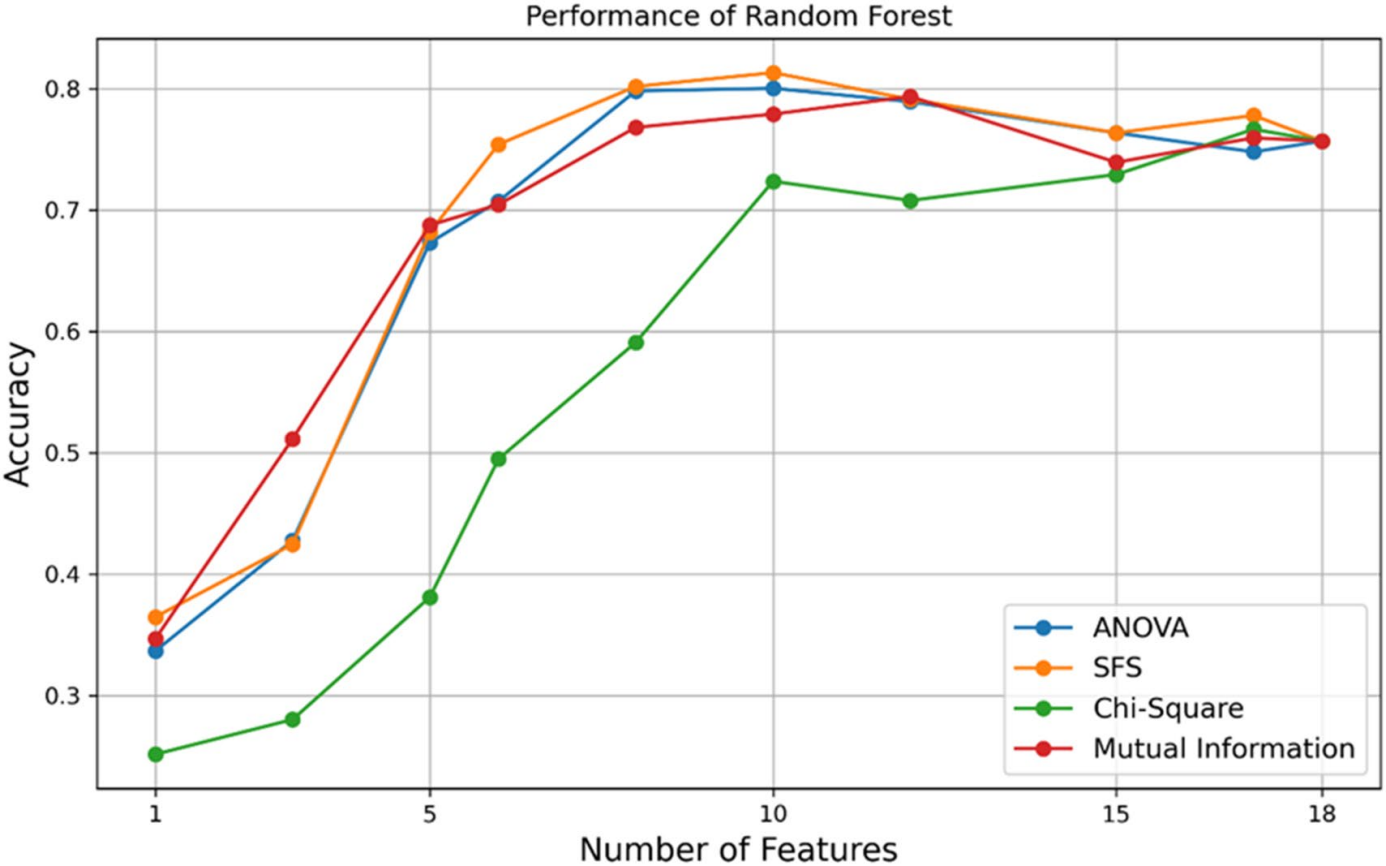
Performance comparison of various feature selection methods for the best-performing classifier, Random Forest. The x-axis represents the number of features, and the y-axis indicates accuracy. SFS feature selection method outperforms others, achieving a peak accuracy of 81.32% with 10 features.

Figure 2 shows the ratio of the number of features selected by all four methods of SFS, MI, Chi-square, and ANOVA to the accuracy obtained with the Random Forest classifier. As can be seen, the highest accuracy has been achieved in all four methods with 10 features. Among these selected features GPT, γ-GTP, TG, BMI, HbA1c, Lp(a), Loge(Lp(a)), HDL, LDL SI, and Ezetimibe, using SFS and the RF classifier algorithm.

## Discussion

The inability to identify NASH swiftly and dependably may result in the progression of conditions such as liver cirrhosis, hepatocellular carcinoma, and a heightened vulnerability to cardiovascular disease. Therefore, prompt diagnosis is essential to reduce future risks for patients and adjust their treatment costs. In this paper, the ability of advanced machine learning algorithms was evaluated to estimate NASH disease based on demographic information and blood tests. Although the performance of all classification algorithms is in the similar tolerance range in the four feature selection methods, the results show that the chosen algorithm achieved a maximum accuracy of 81.32% with 10 selected dominant features, including GPT, γ-GTP, TG, BMI, HbA1c, Lp(a), Loge(Lp(a)), HDL, LDL SI, and Ezetimibe, using SFS and the RF classifier algorithm. The recall index indicates that the proposed algorithm can provide the most confident classification compared to other studied algorithms. Moreover, the f1-score index results in 83.75, indicating that the algorithm has a better ability to diagnose the correct class than other evaluated algorithms. Therefore, the proposed intelligent algorithm can automatically estimate NASH with appropriate performance, provide an initial report of the patient’s situation without invasive modality for specialists, and help them to clarify the diagnosis procedure and, subsequently, the cycle of prevention and treatment. Table 4 compares the proposed approach with other approaches that were proposed in recent years regarding to classification of NASH. This table compares the data count, modality type, machine learning methods used, and the results obtained.

**Table 4:** Comparison of the proposed approach with other approaches in recent years regarding liver fat classification.

Ref	No. of samples	Modality	ML algorithm	Classification	Performance
Ma et al.^10^	10,508 enrolled subjects	Demography records	BN	NASH Non-NASH	ACC 83% Sen 67.5% Spec 87.8% F1-score 65.5%
Newsome et al.^13^	350 patients	Demography records	LR	Stage of NASH	Spec 90% Sen 90% PPV 0.83 NPV 0.85
García Carretero et al.^17^	1525 patients	Demography records	RF	NASH Non-NASH	ACC 87.2% Sen 64.2% Spec 96.6% AUROC 83.7
Zamanian et al.^19^	176 patients	Demography records	RF, LR, LDA, SVM, MLP	NASH Non-NASH	ACC 81.98% AUC 79.68% Pre 85.37% Rec 87.65%
Proposed method	176 patients	Demography records	AdaBoost, LightGBM, LR, RF, SVM, XGBoost	NAFLD activity score	ACC 81.32% Sen 86.04% Pre 81.59% Spec 70.49% F1-score 83.75%

BN: Bayesian network; LR: Logistic Regression; RF: RandomForest; SVM: Support Vector Machine; MLP: Multi-Layer Perceptron.

According to this table, the proposed method demonstrates superiority over previously reported works in evaluating the NAS Score class within a three-class category. It offers a more comprehensive assessment compared to earlier efforts focused on two classes. This classification categorizes disease degree changes as low, middle, and high boundaries of NAS Score. Furthermore, it provides the most accurate measurement possible based on the available data, avoiding overfitting. Both the study by Zamanian et al.^19^ and the proposed method utilized the same dataset, but their classification methodologies differed. The former study utilized a binary classification model to distinguish between healthy livers and those affected by fatty liver conditions in 176 participants, achieving an accuracy of 81.98%. The second study, in contrast, adopted a multiclass approach, classifying participants into three categories based on the NAS, achieving an accuracy of 81.32%. The study^19^ experimented with both linear algorithms such as LR and SVM, as well as non-linear techniques including RF, LDA and MLP. In contrast, the proposed method evaluated a wider array of classifiers, encompassing ensemble methods like AdaBoost, LightGBM and XGBoost in addition to LR, SVM and RF. The incorporation of cutting-edge boosting algorithms is a crucial distinction in the proposed approach. Each classifier within the proposed method underwent a systematic exploration of hyperparameter space to optimize predictive accuracy. This process, detailing the range of hyperparameters for individual classifiers, as illustrated in Table 2, exemplifies a transparent approach aimed at fine-tuning models for enhanced predictive capabilities. Furthermore, the proposed method employed a repeated leave-one-out cross-validation technique, differing from the methodology used in the former study, which could have influenced the models' performance and generalization^33^.

In the proposed structure, we utilized four common algorithms for dominant feature selection, including SFS, ANOVA, Chi-square, and MI, based on their inherent differences. The experiment showed that the range of the performance is similar in all algorithms in an appropriate value. SFS algorithm offers distinct advantages over other feature selection methods. SFS incrementally builds feature subsets by selecting one feature at a time, assessing its impact on model performance. This iterative approach allows SFS to explore a broader feature space efficiently^34^. Unlike ANOVA, where feature selection relies on statistical tests comparing feature means across classes, SFS's method is less influenced by assumptions and limitations^35^. One notable distinction is that SFS avoids the potential for getting trapped in local optima, a concern often encountered by greedy algorithms. This means that SFS is more likely to discover the optimal feature subset that maximizes criteria such as accuracy without prematurely settling for suboptimal choices. Moreover, SFS is not restricted to specific data types or assumptions. Unlike Chi-square, which is only suitable for categorical features and assumes independence between them, and MI, which can be sensitive to noise and outliers, SFS is adaptable to various data types and can accommodate dependencies and noisy data more effectively. This adaptability makes SFS a versatile and robust feature selection method, capable of capturing a wider range of variances and contributing to enhanced model performance. Therefore, SFS offers distinct advantages over other feature selection methods in the context of predicting NASH based on NAS score. One notable advantage of SFS is its ability to systematically build a feature set by iteratively selecting the most relevant features, which can enhance the model's predictive power. Unlike MI and Chi-square, SFS is versatile and can handle both continuous and discrete features, making it suitable for a broader range of data types^36^. However, it is essential to acknowledge that SFS also has its limitations. Like ANOVA, SFS may assume that the sample size is equal for all groups, which may not always hold true in real-world data scenarios. Additionally, SFS, like other feature selection methods, may not be immune to issues related to data distribution and variance equality, which can impact its performance under specific conditions^37^.

The RF algorithm is a robust method that relies on several structural features to achieve a high performance. The comparison of the results shows that using several DT estimators and their majority voting result is inferior to the RF algorithm. The RF applies a bagging technique to samples from the given training dataset to reduce the variation in prediction by applying repeatable combinations in the original data. In other words, the selection of variables in each tree is the criterion for tree design, and their duplication is permitted. Moreover, the RF creates several full-size decision trees with different depths, and the final classification is derived from their assessment. The weights of each decision are also uniform for all trees in the RF. Finally, another feature of the RF algorithm is that the trees are independent from each other.

Although this study highlights the potential of machine learning in diagnosing NASH, some limitations should be acknowledged. First, the data set included only 176 patients from one institution, which may limit the generalizability and robustness of the model. Machine learning algorithms benefit from larger, more varied data to learn effectively and avoid overfitting on narrow patterns. Our modestly sized cohort increases susceptibility to outliers and variability between training and validation performance. Though we utilized cross-validation techniques to reduce overfitting, model accuracy, and portability would be enhanced through expanded multi-center datasets. Secondly, the feature set available for analysis may have influenced model performance. Although we expanded the original 19 input variables to 54 features through preprocessing, the algorithms’ capacity to uncover predictive relationships was still limited compared to studies leveraging extensive omics data. While restrictions can help avoid overfitting, integrating a wider range of clinical lab tests, imaging indicators, and molecular biomarkers could potentially boost diagnostic precision and generalization. Finally, external validation on entirely new data is necessary to truly gauge real-world viability. Finally, external validation on entirely new data is necessary to truly gauge real-world viability. As NASH prevalence and risk factors may differ across populations, applying these models to diverse unpublished datasets will better demonstrate robustness. Through rigorous validation across large patient cohorts from varying demographics and clinics, the generalizability and clinical usefulness of the machine learning approach can be firmly established. Addressing these limitations represents an opportunity for the meaningful progression of this research. Expanding our algorithms' inputs, training data size and diversity, and testing on disparate populations will further strengthen confidence in the use of AI for non-invasive NASH diagnosis and screening.

In future work, we aim to explore the use of computer vision techniques to enhance the diagnosis of NASH. Specifically, we plan to transform the tabular data into images and use data augmentation or generative adversarial networks (GANs) to generate synthetic images that can augment the training data^38,39^. By generating data, we hope to overcome the limitations of small and imbalanced datasets and improve the generalization and robustness of our models. Furthermore, we will apply various image processing and feature extraction methods to both the newly generated images and the synthetic ones. We will then compare their performance with the existing features. We believe that this innovative approach will open up new insights and opportunities for the application of artificial intelligence in non-invasive NASH diagnosis and screening.

## Conclusion

In conclusion, this study demonstrates the potential of using machine learning techniques for the early and non-invasive diagnosis of non-alcoholic steatohepatitis (NASH). We evaluated a range of sophisticated algorithms, including SVM, RF, AdaBoost, LightGBM, and XGBoost, paired with rigorous feature selection methods. Our findings reveal that the optimal combination of SFS for feature selection and Random Forest for classification can predict NASH with high accuracy (81.32%), recall (86.04%), specificity (70.49%), precision (81.59%), and F1-score (83.75%) based on readily available clinical data and blood parameters.

The promising results underscore machine learning's capabilities in augmenting and potentially replacing invasive diagnostic procedures like liver biopsy that are currently considered the gold standard for confirming NASH. Our approach relied solely on demographic information, medication history, and blood test factors, avoiding the risks and discomfort associated with tissue sampling. Furthermore, the non-invasive nature allows for frequent testing and continuous monitoring, enabling early diagnosis and timely treatment before advanced disease stages. However, our study has some limitations, including the small dataset size which may limit the model's generalizability. Future research should focus on expanding to multi-center data encompassing wider demographics and disease severities. Further algorithmic refinements, like generating synthetic training data and extracting visual features, could also help overcome limitations of small imbalanced datasets. Overall, this research demonstrates machine learning's immense potential in addressing a critical medical challenge—the early diagnosis of NASH. Our proposed solution can form the basis for developing reliable non-invasive diagnostic tests that can be applied widely for screening and monitoring at-risk populations. With further research, machine learning promises to transform NASH diagnosis, leading to improved clinical outcomes and reduced disease burden.

## Data Availability

The Proprietary dataset used and analyzed during the current study is available from the corresponding author on reasonable request.
